# Risk factors for robot-assisted spinal pedicle screw malposition

**DOI:** 10.1038/s41598-019-40057-z

**Published:** 2019-02-28

**Authors:** Jia Nan Zhang, Yong Fan, Ding Jun Hao

**Affiliations:** Department of Spine Surgery, Hong Hui Hospital, Xi’an Jiaotong University, No. 76 Nanguo Road, Nanshao gate, Xi’an, 710054 Shaan’xi Province China

## Abstract

The accuracy of robot-assisted pedicle screw placement is unstable and remains controversial. The purpose of this study was to determine the risk factors for unsatisfactory Renaissance robot-assisted pedicle screw placement. This was a retrospective study of prospective data. From January 2017 to March 2018, 136 robot-assisted pedicle screw placements were performed in our department for spinal diseases, and a total of 874 screws were evaluated. All screws were assessed by the Gertzbein and Robbins classification. A and B were defined as satisfactory. C, D, and E were defined as unsatisfactory. Intraoperative registration failures due to nontechnical reasons or intraoperative adjustment were also defined as unsatisfactory. According to the evaluated results, the screws were divided into the satisfactory group (Group A) and the unsatisfactory group (Group B). The satisfactory rate was defined as satisfactory screws (the screws in Group A)/total screws, and the accurate rate was defined as accuracy screws (the screws in Group A)/the screws implanted by the robot (total screws - failed registration screws - screws adjusted during the operation). The age, sex, BMI, and BMD as well as the type of disease, the degree of vertebral rotation and the type of screw placement (percutaneous implantation or open implantation) were compared between the two groups, with the assessment of potential risk factors for unsatisfactory robot-assisted screw placement using logistic regression. A total of 874 screws were evaluated; there were 759 screws in Group A and 115 screws in Group B. The satisfactory rate was 86.8% (759/874), and the accuracy rate of the robot-placed screws was 94.4% (759/804). After logistic regression analysis, the independent risk factors were identified as obesity (OR 5.357 [95% CI 2.897–9.906], p < 0.01), osteoporosis, vertebral rotation and the presence of congenital scoliosis (OR 9.835 [95% CI 4.279–22.604], p < 0.01), particularly for severe osteoporosis (T < −3.5) and severe vertebral rotation (III-IV). According to the results of this study, obesity, osteoporosis and congenital scoliosis are risk factors for unsatisfactory robot-assisted screw placement. Furthermore, for surgeons in the initial stage of using a robot, we suggest avoiding cases in which a single risk factor or multiple risk factors exist to ensure the safety of the operation and to help augment the confidence of the surgeons.

## Introduction

Since it was invented, the technique of pedicle screw fixation has been widely used in spinal fractures, spinal degenerative diseases, etc.; it is a milestone in the development of spinal surgery. However, there are some risks and complications with pedicle screw fixation due to the adjacent nerve roots, spinal cord and blood vessels around the pedicle^1^. Therefore, the key to the success of the pedicle screw fixation technique is to improve the accuracy of pedicle screw fixation. With the development of medical imaging technology and computer technology, image navigation and robot technology have been gradually applied to pedicle screw placement. It has been reported that robot-assisted pedicle screw placement has higher accuracy, lower radiation exposure and fewer complications in some studies^2–5^. However, the accuracy of robot-assisted pedicle screw placement is unstable and remains controversial. A prospective randomized study showed that robot-assisted pedicle screw placement did not have better accuracy of screw placement^6^. A meta-analysis by Marcus *et al*. also showed that robot-assisted placement did not show higher accuracy than free hand placement^4^. These results may be due to differences in the diseases. The Renaissance robot system is the first technique approved by the FDA for robot-assisted spinal surgery^2,7^. Global spine surgeons were widely concerned about the accuracy and safety of this system. However, while the focus of the previous studies was to evaluate the accuracy of the screws, few studies have focused on the successful performance of robot-assisted screw placement. The spinal surgical robot is a semi-autonomous robot, and most steps of the procedure require manual operation by the surgeon. This procedure may affect the accuracy of the screw because of differing conditions. With the increase in use of the application, many scholars have noticed that several factors may affect screw placement. However, as far as we know, there has been no research focused on the risk factors that affect the failure or inaccuracy of robot-assisted placement. This study was a retrospective analysis of the risk factors for failure of Renaissance (Renaissance™, Mazor Robotics, Caesarea, Israel) robot-assisted pedicle screw placement.

## Materials and Methods

### Selection of patients

This was a retrospective study of prospectively collected data. From January 2017 to March 2018, 136 robot-assisted pedicle screw placements (male: 74, female: 62) were performed in our department for spinal diseases, and a total of 874 screws were evaluated. All cases were selected from 3 surgical teams trained by simulated robot-assisted screw placement, and the first 10 cases were excluded for each team. X-ray, CT (1 mm thickness scan, sagittal and coronal reconstruction) and MRI examinations were performed before the operation. The preoperative examination showed no obvious contraindications as well as definite operative evidence. The age, sex, BMI (BMI < 27 was defined as not obese, BMI ≥ 27 was defined as obese), and BMD (T > −2.5, 3.5 < T ≤ −2.5 and T ≤ −3.5) as well as the type of disease (including congenital scoliosis, spinal lumbar degeneration diseases, fracture, adolescent idiopathic scoliosis, AIS, etc.), whether or not there was revision surgery, the degree of vertebral rotation (Nash-Moe’s method; 0 was defined as no rotation, I-II was defined as mild rotation, and III-IV was defined as severe rotation) and the type of screw placement (percutaneous implantation or open implantation) were compared between groups to assess potential risk factors.

**Figure 1:**
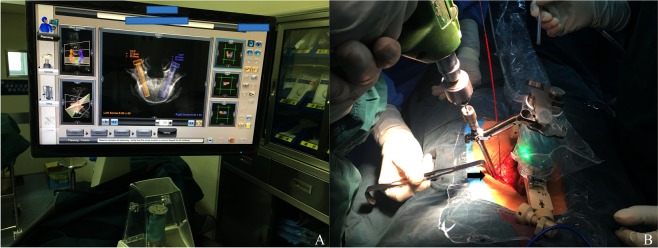
A plan for the screw trajectories for a lumbar revision surgery on the workstation. (**A**) This was an obesity patient who underwent lower lumbar revision surgery. As the soft tissue was thickening, we had to place the fascial screw from a new incision, and half of the screws (3/6) were adjusted during the operation due to unsatisfactory screw placement (**B**).

Surgical procedures:

1. The preoperative plan was based on the optimal location and length of the screws in the preoperative CT scan plan. The preoperative plan was performed by the surgeon and the assistant surgeons to ensure that the design was reasonable.
2. After the patient was anaesthetized, the robot workstation was connected to the data line of the C-arm X-ray machine (Arcadis Orbic 3D, Siemens, Henkestr, Germany), and the CT data from the patient were imported. The clip or the fixed needle was used to install the metal tag attachment to the correct position. Some cases needed to be adjusted and positioned several times.
3. The intraoperative fluoroscopy image (AP and oblique images) data from the C-arm were registered and matched with the preoperative CT data. On the robot computer, the screw placement points and the needle angles were adjusted appropriately.
4. When the appropriate “bridge” was selected and installed to ensure stability, the robot was installed on the back of the patient’s back support, and the proper installation of the specified manipulator was selected according to the preoperative planning and the robotic manipulator; then, the channel on the robot was selected according to the robot computer. The robot adjusted the position automatically according to the screw which needed to be implanted; then, the surgeon bored a hole along the channel and placed the needle.
5. The AP and lateral images from the C-arm were performed, and the surgeon determined whether the position of the needle should be adjusted or not according to experience. For the open operation, a manual adjustment of the screw canal was performed. The pedicle screw was placed after the safety of the screw canal was confirmed. For the percutaneous cases, the screw, which needed to be adjusted, was implanted under fluoroscopy guidance (Fig. 1).

### Postoperative care

After the removal of the drainage tube, a CT scan was performed on the fixed segments to assess the accuracy of each screw (except for the manually implanted screws). All the screws were assessed by the Gertzbein and Robbins classification with the following grades: A, the cortical layer of the pedicle is complete; B, C and D, penetrates the cortical layer of the pedicle but is less than 2 mm, 4 mm and 6 mm, respectively; E, breaches the cortical layer of the pedicle in any direction by more than 6 mm or is outside of the pedicle^8^. A and B were defined as satisfactory. C, D, and E were defined as unsatisfactory. Intraoperative registration failure due to non-technical reasons or due to intraoperative adjustments were also defined as unsatisfactory. According to the evaluated results, the screws were divided into the satisfactory group (Group A) and the unsatisfactory group (Group B). The satisfactory rate was defined as satisfactory screws (the screws in Group A)/total screws, and the accurate rate was defined as accuracy screws (the screws in Group A)/the screws that were implanted by the robot (total screws - failed registration screws - screws were adjusted during the operation). A surgeon on our research team collected radiological data; however, radiological data were evaluated by two other surgeons, and if the results of the two evaluators were inconsistent, the final decision was made according to the opinion of the third surgeon.

This work was supported by the National Key Research and Development Plan of China (Precision treatment solution of high-risk complex orthopaedic diseases Grant No. 2016YFC0105804). We confirmed that all methods were carried out in accordance with relevant guidelines and regulations of our licensing committee. We confirmed that all experimental protocols were approved by the licensing committee of Xi’an HongHui Hospital and that informed consent was obtained from all patients. Informed consent for publication of identifying images has been approved by each relevant patient

### Statistical analysis

Statistical analyses were performed with SPSS 22.0 software (SPSS Inc., Chicago, Illinois). The chi-squared test was used for analysis of intergroup differences, and the Student’s t-test for independent samples was used to compare the two sets of data that were normally distributed. P < 0.05 was considered statistically significant. The significance of each of the potential risk factors was assessed using the stepwise logistic regression test. P < 0.05 was considered statistically significant.

## Results

A total of 874 screws were evaluated, and 39 screws were adjusted during the operation. There were 31 failed registration screws in total, and 19 screws could not be registered in 2 patients with congenital scoliosis. There were 45 C, D and E screws evaluated after the operation. There were 759 screws in Group A and 115 screws in Group B. The satisfactory rate was 86.8% (759/874), and the accuracy rate of the robot-placed screws was 94.4% (759/804).

The chi-squared test showed no significant difference for sex (p = 0.07); however, other variables had significant differences (Table 1). The variables for age, BMI, and BMD, congenital scoliosis, spinal lumbar degeneration diseases, fracture, adolescent idiopathic scoliosis, AIS, whether or not there was revision surgery, degree of vertebral rotation and type of screw placement were included in the stepwise logistic regression analysis. After univariate analysis, the included factors were identified as significantly different between the two groups (P < 0.05); sex was excluded because there was no significant difference between the two groups (p = 0.07).

**Table 1:** Univariate analysis of unsatisfactory of Renaissance robot-assisted pedicle screw placement.

Variable	Group A	Group B	Value P*
Age(years old)			
<65	538	65	P < 0.05^#^
>65	221	50	
Sex(male/female)	408/351	51/64	P = 0.07
BMD(T-score)			
>−2.5	566	65	P < 0.05^#^
−3.5 < T ≤ −2.5	139	22	
T ≤ −3.5	54	28	
BMI(kg/m^2^)			
<27	486	50	P < 0.05^#^
≥27	388	65	
Vertebral rotation			
0	576	53	P < 0.05^#^
I–II	175	45	
III–IV	8	17	
Diagnosis			
Congenital scoliosis	63	34	P < 0.05^#^
Degenerative scoliosis	59	24	
Lumbar degeneration diseases	200	31	
AIS	89	7	
Fracture	348	19	
Percutaneous approach	339	20	P < 0.05^#^
Revision surgery	50	32	P < 0.05^#^

*Chi-square test.

^#^Indicates significance.

In the stepwise logistic regression analysis, the independent risk factors identified by stepwise logistic regression were obesity (OR 5.357 [95% CI 2.897–9.906], p < 0.01), osteoporosis, vertebral rotation and the presence of congenital scoliosis (OR 9.835 [95% CI 4.279–22.604], p < 0.01), especially for severe osteoporosis (T < −3.5) and vertebral rotation (III-IV) (Table 2).

**Table 2:** Analysis of risk factors by logistic regression test.

Variable	OR (95% CI)	Value P*
BMD(T-score)		
−3.5 < T ≤ −2.5	4.615 (2.269~9.386)	P < 0.01^#^
T ≤ −3.5	8.805 (4.650~16.673)	P < 0.01^#^
BMI(kg/m^2^)		
≥27	5.357 (2.897~9.906)	P < 0.01^#^
Vertebral rotation		
I-II	6.724 (2.514~17.985)	P < 0.01^#^
III-IV	14.004 (4.881~40.182)	P < 0.01^#^
Diagnosis		
Congenital scoliosis	9.835 (4.279~22.604)	P < 0.01^#^

*Logistic regression.

^#^Indicates significance.

Only two patients had radiative pain in the lower extremity due to the screws, the symptoms of which disappeared after conservative treatment, and no infection or severe nerve or vascular injuries were found in this study.

## Discussion

Since the pedicle screw fixation technique became widely used, spinal surgeons have paid increasing attention to the accuracy of pedicle screw placement. With the application and development of robot-assisted technology in spine surgery, the advantages of high stability and high accuracy have gradually been reported in some studies. Nevertheless, the accuracy rates were quite different in previous literature, and the accuracy of robot-assisted pedicle screw placement was 90–98%^3,9,10^. Currently, the robot remains an assistance tool, a semi-autonomous robot, and the operation mode is the ‘Robot + Surgeon + Patient’. The robot is stable when the operation of the system is correct, and the previous study also showed that when the surgeon was familiar with the operation process and had finished a certain number of robot-assisted operations, the accuracy of screw placement was not affected by surgeon interference^11^. Therefore, the related factors of the patient are the main factors that may interfere with the success and accuracy of screw placement. With the attention on the clinical application of the robot, researchers have noticed some possible risk factors that may influence the application of the robot but, these have not been confirmed by specific research.

As a retrospective study, when we found that some factors may influence the implantation of screws, we decided to collect and analyse relevant data. According to experience, the necessary sample size can be simply estimated as the number of variables multiplied by 10. Following our data collection, there were 18 variables and 874 screws in our study; 874 is far greater than 180 (18 × 10 = 180). We analysed sample sizes for every factor, and found that except for severe rotation (III-IV) with 25 screws, the sample size of every other factor was more than 80 screws. At the same time, we reviewed previous studies of robot-assisted spinal surgery and found that our study had larger sample sizes of patients and screws. We determined that the present sample size has enough power to support the conclusions of this study.

### Key results

This study included all the screws that were adjusted intraoperatively or that experienced registration failure due to non-technical reasons. The results showed that the satisfactory rate of pedicle screw placement was 86.8% (759/874) and that the accuracy rate of the robot-placed screw was 94.4% (759/804); the robot still showed excellent results. The independent risk factors identified were osteoporosis (−3.5 < T ≤ −2.5 OR 4.615 [95% CI 2.269~9.386]; T ≤ −3.5 OR 8.805 [95% CI 4.650~16.673]), obesity (BMI ≥ 27 kg/m^2^ OR 5.357 [95% CI 2.897–9.906]), and vertebral rotation (I-II OR 6.724 [95% CI 2.514~17.985]); III-IV 14.004 [95% CI 4.881~40.182]) as well as the presence of congenital scoliosis (OR 9.835 [95% CI 4.279–22.604]). We found that inaccurate placement was more common in obese patients, especially for lower lumbar surgery. Potential reasons for this outcome may be that the soft tissue was too thick and that the tissue tension was high. In patients with soft congenital scoliosis, the robot occasionally failed at the step of registration. The reasons for this may be related to muscle relaxation after anaesthesia, which may have caused a difference between the intraoperative position and the preoperative CT scan. We found that in the condition of severe rotation of the vertebral body, with robot assistance, some screws were difficult to place because of the limited range of the robot and the shielding of surrounding tissues. At the same time, poor bone quality (lower BMD) may lead to the failure of matching.

### Comparison with other studies

The use of new technology always involves a learning curve before reaching a steady state. Therefore, we reviewed previous studies of robot-assisted spinal surgery or computer-assisted spinal surgery. Those studies found that approximately 10–30 surgeries were performed before the surgeon became skillful^11–13^. However, some studies were different from the robot system used in our study; the actual learning curve for use of spinal robots may vary among systems. Hyun *et al*.^5^ found there was no change in accuracy after the first 5 clinical cases using the same robot system as this study. In our experience, surgeons can perform the robot-system proficiently after approximately 5–10 surgeries. Therefore, combining the previous study findings with our experience, we chose to ignore the first 10 cases.

Some previous studies have reported that factors such as BMD and BMI may be causes for the inaccuracy of robot-assisted placement and the failure of registration^14,15^. Our study found that some osteoporotic patients had poor bone quality; although the Peteron technique could handle the screw entry point^16^, some needles could still not be properly fixed on the bone, and a slight jitter led to the deviation of the occlusal point. At the same time, poor bone quality (lower BMD) may lead to the failure of matching. Tsai *et al*. also noted that the robot has a high requirement for body position; compared with the preoperative CT scan, the intraoperative body position cannot be changed too much because otherwise it is difficult to match^17^. Previous studies have found that there was a problem of “casing slip” on the surface of a slope, which led to unsatisfactory screw placement^10,18^. One research study also found that cases of abnormally steep slope could give rise to a lateral skidding of the cannula at the screw entry point due to the un-consistently reliable anchorage of the cannula^6^. Moreover, such patients are older, which often increases the risk of inaccurate placement when combined with osteoporosis and obesity. Hu *et al*.^19^ reviewed 102 cases with robot-assisted screw placement. They found that 89.5% of the patients had spinal deformity or needed revision surgery, and the results showed that the real success rate of the screw placement was 87.5% (949/1085), with as many as 110 screws replaced manually in the robot-assisted cases and 15 screws abandoned. In our initial cases, we found that in patients who underwent open surgery, especially those with thick soft tissue of the back, it was sometimes difficult to keep the channel led by the robot in the wound, and the surgeon often had to prolong the incision for percutaneous screw placement. At the same time, for cases undergoing lower lumbar revision surgery combined with obesity, the above situation would also occur; because of the outer screw entry point and larger angle of the screw, we had to elongate the incision or place a percutaneous screw from a new incision. However, both approaches added unnecessary injury, especially where accuracy and minimal invasiveness are required (Fig. 1). In a prospective randomized study by Ringel *et al*., it was found that for the accuracy of screw placement, the robot did not show an advantage in placing lower lumbar and sacral screws^6^. Upon review of the literature, we found that most of the articles did not seriously discuss this problem, and these failed cases were also excluded from the screw evaluation^3,19^. We believed this exclusion was not conducive to the correct application of the robot and that it greatly reduced the guiding meaning for peers. The robot-assisted technique has brought some changes to spine surgery; however, this technique still has some limitations. We need to choose appropriate cases to utilize the advantages of robots. The purpose of this study was to determine the related risk factors for unsatisfactory robot-assisted screw placement. Furthermore, for surgeons in the initial stage of using a robot, we suggest avoiding cases in which a single risk factor or multiple risk factors exist to ensure the safety of the operation and to help augment the confidence of the surgeons.

### Strategies

In our experience, for cases with severe vertebral rotation, the robot-assisted placement application should be avoided and computer-navigation assisted placement is a better choice. For patients with higher BMIs and thick soft tissue of the back, the incision should be lengthened appropriately and the assistant should pay additional attention to protecting the operation of screw implantation. At the same time, for complex cases, multiple technologies can be used simultaneously. Exploiting the advantages and avoiding the weaknesses is the key to efficient utilization of any technology.

### Limitations

This study still has some limitations. First, this was a single-centre, retrospective study; so there is a need for a multicentre prospective study with a larger sample size to verify our findings. Second, our study included three different surgeon groups. However, all the surgeons were trained before performing operations, and the study excluded the first 10 cases in each group. Finally, due to limited sample size, several factors, such as malformed vertebrae, that may contribute to unsatisfactory pedicle screw position have not been assessed in the study.

## Conclusion

According to the results of this study, robot-guided pedicle screw placement is a well-established technique, and we found that obesity, osteoporosis and congenital scoliosis are risk factors for unsatisfactory robot-assisted screw placement. We suggest that it is better to avoid cases containing the above risk factors in the early stages of robot applications.

## References

[1] Lonstein JE (1999). Complications associated with pedicle screws. J. Bone Joint Surg. Am..

[2] Pechlivanis I (2009). Percutaneous placement of pedicle screws in the lumbar spine using a bone mounted miniature robotic system: first experiences and accuracy of screw placement. Spine.

[3] Devito DP (2010). Clinical acceptance and accuracy assessment of spinal implants guided with SpineAssist surgical robot: retrospective study. Spine.

[4] Marcus HJ, Cundy TP, Nandi D, Yang GZ, Darzi A (2014). Robot-assisted and fluoroscopy-guided pedicle screw placement: a systematic review. Eur. Spine J..

[5] Hyun SJ, Kim KJ, Jahng TA, Kim HJ (2017). Minimally Invasive Robotic Versus Open Fluoroscopic-guided Spinal Instrumented Fusions: A Randomized Controlled Trial. Spine.

[6] Ringel F (2012). Accuracy of robot-assisted placement of lumbar and sacral pedicle screws: a prospective randomized comparison to conventional freehand screw implantation. Spine.

[7] Barzilay Y (2014). Robot-assisted vertebral body augmentation: a radiation reduction tool. Spine.

[8] Gertzbein SD, Robbins SE (1990). Accuracy of pedicular screw placement *in vivo*. Spine.

[9] Keric N (2017). Evaluation of surgical strategy of conventional vs. percutaneous robot-assisted spinal trans-pedicular instrumentation in spondylodiscitis. J. Robot. Surg..

[10] Schatlo B (2014). Safety and accuracy of robot-assisted versus fluoroscopy-guided pedicle screw insertion for degenerative diseases of the lumbar spine: a matched cohort comparison. J. Neurosurg. Spine.

[11] Hu X, Lieberman IH (2014). What is the learning curve for robotic-assisted pedicle screw placement in spine surgery?. Clin. Orthop. Relat. Res..

[12] van Dijk JD, van den Ende RP, Stramigioli S, Kochling M, Hoss N (2015). Clinical pedicle screw accuracy and deviation from planning in robot-guided spine surgery: robot-guided pedicle screw accuracy. Spine.

[13] Lee MH (2013). Feasibility of Intra-operative Computed Tomography Navigation System for Pedicle Screw Insertion of the Thoraco-lumbar Spine. Journal of spinal disorders & techniques.

[14] Tian W (2014). A robot-assisted surgical system using a force-image control method for pedicle screw insertion. Plos One.

[15] Roser F, Tatagiba M, Maier G (2013). Spinal robotics: current applications and future perspectives. Neurosurgery.

[16] Kim, H. J. *et al*. A prospective, randomized, controlled trial of robot-assisted vs freehand pedicle screw fixation in spine surgery. *Int*. *J*. *Med*. *Robot*. **13**, 10.1002/rcs.1779 (2017). 10.1002/rcs.1779 27672000

[17] Tsai TH (2017). Pedicle screw placement accuracy of bone-mounted miniature robot system. Medicine (Baltimore).

[18] Molliqaj G (2017). Accuracy of robot-guided versus freehand fluoroscopy-assisted pedicle screw insertion in thoracolumbar spinal surgery. Neurosurg. Focus.

[19] Hu X, Ohnmeiss DD, Lieberman IH (2013). Robotic-assisted pedicle screw placement: lessons learned from the first 102 patients. Eur. Spine J..

